# The Impact of Early-Life Growth on Long-Term Cardiometabolic and Neurocognitive Outcomes in High-Income Countries: A Neglected Public Health Problem

**DOI:** 10.1093/nutrit/nuaf098

**Published:** 2025-07-09

**Authors:** Joseph Freer, Joanna Orr, Jonathan C K Wells, Andrew J Prendergast

**Affiliations:** Centre for Genomics and Child Health, Blizard Institute, Queen Mary University of London, London, United Kingdom; Centre for Genomics and Child Health, Blizard Institute, Queen Mary University of London, London, United Kingdom; Great Ormond Street Institute of Child Health, Population, Policy & Practice Department, University College London, London, United Kingdom; Centre for Genomics and Child Health, Blizard Institute, Queen Mary University of London, London, United Kingdom; Zvitambo Institute for Maternal and Child Health Research, Harare, Zimbabwe

**Keywords:** stunting, child growth, child development, life course, capacity-load model, noncommunicable disease epidemiology

## Abstract

Stunting affects approximately one-quarter of children worldwide. While the majority of these children live in Africa and Asia, a social gradient in height exists within high-income countries (HICs). In contrast to the coordinated focus on stunting in many low- and middle-income countries (LMICs), linear growth is not a public health priority in high-income settings. We reviewed the literature on relationships between linear growth and cardiometabolic outcomes (coronary heart disease [CHD], overweight/obesity, hypertension, and type 2 diabetes) and neurocognitive outcomes. We take a life-course approach, and use the “capacity load” model as a framework for understanding why stunting is associated with adverse cardiometabolic outcomes. We focus on the literature from HICs, but data from LMICs are included for context. Analysis of birth cohorts in high-, middle-, and low-income countries has consistently demonstrated relationships between early-life linear growth and downstream cardiometabolic and neurocognitive outcomes. These findings have been reinforced more recently by trial data. Birth size is associated with CHD, obesity, and type 2 diabetes, and with hypertension when small birth size is followed by accelerated postnatal growth. The patterns of postnatal linear growth associated with outcomes in adulthood are complex and context-dependent; both stunting and rapid linear growth in childhood are associated with cardiometabolic disease. We interpret these findings using the capacity-load model, a conceptual framework that describes the interactions between metabolic homeostatic capacity and metabolic load in shaping CHD risk. The strength and consistency of longitudinal associations between linear growth and several long-term deleterious outcomes across multiple settings and time points are striking. Future research should investigate both causal pathways and the potential for using height and growth velocity as markers for identifying children at increased risk of lifelong decrements in educational or cardiometabolic health outcomes who could benefit from early intervention.

## INTRODUCTION

Stunting affects approximately 150 million children under 5 years old—approximately one-quarter of children worldwide.^1^^,^^2^ Defined as impaired linear growth that has resulted in a height-for-age more than 2 SDs below the population median, stunting is a consequence of the multiple factors that determine growth in early life. For most children experiencing stunting globally, it is a physical manifestation of an adverse environment, occurring in the context of economic and psychosocial disadvantage. The World Health Organization (WHO) conceptual framework for stunting encompasses overlapping proximal factors, including infection, nutrition, water and sanitation, and the distal determinants of society, economy, education, and healthcare.^3^

Vast and persisting global North–South inequalities in these factors mean that the majority of children experiencing stunting live in Africa and Asia.^1^ However, a social gradient in stunting exists not just between, but also within, countries; a recent analysis of national data from England identified a monotonic relationship between deprivation and short stature, amounting to a 4-fold regional variation in stunting prevalence.^4^ In spite of the clear relationship between poverty and height, linear growth is not currently a public health priority in most high-income countries (HICs). Indeed, while the term “stunting” is in common usage in the public health discourse in low- and middle-income countries (LMICs) and its definition incorporates factors related to poverty,^3^ in HICs, children without an identifiable underlying biological cause of linear growth faltering are usually instead labelled as having “idiopathic short stature.” This diagnosis often signals an end, rather than a beginning, to investigation and intervention—a “medicalized” approach that potentially overlooks the psychosocial environment that ultimately drives stunting.

This is in stark contrast to the coordination and intensity of the academic, political, and public health focus on linear growth in many LMICs, which is grounded in evidence in these settings of relationships between poverty, linear growth, neurodevelopment, and long-term cardiometabolic health.^5^ While the prevalence of stunting is lower in HICs, social inequalities in educational outcomes and morbidity and mortality from cardiometabolic disease are wide (and in many cases widening).^6^^,^^7^ Moreover, coronary heart disease (CHD) is the leading cause of death and loss of disability-adjusted life-years from a noncommunicable disease.^8^ A better understanding of the etiology of these outcomes—including the relationships, if any, with early-life linear growth in HICs—is central to improving population health and reducing health inequalities.

## METHODS

We reviewed the literature on relationships between linear growth and several downstream outcomes: cardiometabolic outcomes (CHD, overweight/obesity, hypertension, and type 2 diabetes [T2D]) and neurocognitive outcomes. We focused mainly on the literature from HICs but data from LMICs are included for context and comparison. We searched Medline using the terms “stunting,” “short stature,” “child growth,” “linear growth,” “height,” “heart disease,” “cardiovascular disease,” “coronary heart disease,” “ischaemic heart disease,” “diabetes mellitus,” “hypertension,” “blood pressure,” “weight,” “body mass index,” “overweight,” “obesity,” “chronic disease,” “morbidity,” “mortality,” “neurodevelopment,” and “cognitive development” for studies published since January 1, 1980, with no language restrictions. We also screened the reference lists of studies identified, and searched the gray literature.

## CONCEPTUAL FRAMEWORK

In this review, we took a life-course approach, interpreting the literature on associations between linear growth and cardiometabolic/neurocognitive outcomes both across a lifetime from conception and from a multigenerational perspective. We synthesized the literature to answer the following: (1) “How?”—What are the mechanisms that underlie relationships between growth and long-term deleterious outcomes; (2) “Why?”—What are the conceptual models that explain the growth/disease trade-offs made within a body, a mother–child pair, or across multiple generations; and (3) “When?”—What are the stages of growth over the life course that drive associations with long-term outcomes, and how do they (eg, intrauterine and pubertal growth) relate to each other in the development of disease. We describe and build on the “growth acceleration” and “thrifty phenotype” hypotheses using the lens of the capacity-load model, a conceptual model that we believe explains most clearly the complex relationships between poverty, nutrition, growth, and cardiometabolic disease.^2^

## CAUSALITY

The majority of studies examining associations between growth and cardiometabolic disease are observational, with effect estimates liable to imperfect control for confounding. It is possible that growth trajectories and/or height measured at any one time share a common set of factors with the pathophysiology of cardiometabolic disease, while growth itself may not be on the causal pathway between those factors and chronic disease. Viewed in this way, stunting could be just a marker of factors in early life that result in chronic disease. If this is the case, while stunting could be a useful predictor of long-term disease outcomes (and therefore used to identify individuals at higher risk), prevention or reversal of stunting per se would not prevent disease. To guide the public health response to stunting in HICs, this is a crucial distinction. Where available, we have included randomized trials, including experimental evidence from nutritional supplementation trials and studies that used Mendelian randomization. Each section of the article includes a review of the potential mechanisms underlying the relationships between growth and chronic disease/developmental outcomes, highlighting pathways on which growth and long-term outcomes might be causally linked in high-income settings.

Associations between growth and cardiometabolic disease are particularly complicated by relationships with socioeconomic position (SEP), which is strongly associated with cardiometabolic disease,^9^ as well as with preterm birth,^10^^,^^11^ small-for-gestational age (SGA),^12^ and (independently from prematurity and SGA) with stunting in childhood,^4^^,^^13^ pubertal development,^14^ and short final attained height in adulthood.^15^ Not all observational studies included in the review adequately controlled for SEP, which is difficult to define and categorize, and can vary across the life course.^16^ This is especially problematic for birth cohort studies, which often utilize parental characteristics as proxies for an individual’s lifelong SEP. Furthermore, over successive birth cohorts, the relationship between SEP and body mass index (BMI; body mass divided by the square of height) has reversed in most HICs, changing from a positive association in the early to mid-20th century to a negative association in the late 20th and early 21st centuries.^17^ This is important because BMI and height often have opposite associations with cardiometabolic outcomes, and these anthropometric measures have changing relationships with each other across the life course (height and BMI tend to be positively correlated in childhood and negatively correlated in adulthood).^18^ Notwithstanding these complexities, associations between growth or cardiometabolic disease at various points during life do often persist after careful adjustment for SEP in childhood and adulthood (separately and/or simultaneously). We draw attention to approaches to adjustment for SEP in papers cited throughout the review.

## STUNTING AND NORMAL PATTERNS OF GROWTH

The expected pattern of linear growth for a child born at term, with a normal birth weight, into an unconstrained environment involves rapid length gain in the first 12 months of life, followed by a deceleration until approximately 2–3 years of age, from which point growth velocity is relatively stable until adolescence.^19^ Growth slows just before the onset of puberty and then rapidly accelerates in mid-puberty.^19^ The 2006 WHO Multicentre Growth Reference Study found that breastfed infants and children of nonsmoking mothers living in economically advantaged environments have very similar growth patterns around the world,^20^ and the INTERGROWTH-21st study (International Fetal and Newborn Growth Consortium for the 21st Century) demonstrated that this is also true of fetal and neonatal growth.^21^

Outside of these optimal contexts, stunting typically begins in utero*—*resulting in a low birth weight and short birth length—and in many LMICs, children’s height-for-age continues to decline until at least 2 years of age,^22^ and possibly for much longer.^4^^,^^5^^,^^23^^,^^24^ Accelerated growth of stunted children is sometimes described as “recovery”; this is relatively common in HICs, but there is limited potential for recovery among stunted children living in conditions of marginal diets and persistent poverty.^23^^,^^25–27^ In HICs, the pattern of postnatal growth depends on the etiology of small size at birth, the socioeconomic environment in which children grow up (and therefore the era and setting in which the study was conducted), and the extent/type of nutritional supplementation in infancy.^28^^,^^29^ Children born SGA (birth weight or length <10th centile) often experience growth acceleration in infancy (usually defined as a growth velocity above the median).^30^ Thereafter, both preterm (<37 weeks’ gestation) and term SGA children tend to remain shorter and lighter than term children born appropriate-for-gestational age (AGA).^28^^,^^31^ Infants born preterm are often substantially lighter and shorter when reaching term-equivalent age than infants born at term.^32^ After an initial postnatal period of growth faltering, preterm infants tend to experience accelerated growth, which often continues into early childhood.^32–34^ Growth acceleration in infancy depends also on birth length. For example, data from the Infant Health and Development Program in the United States demonstrated that preterm SGA infants born with low length-for-age tended to experience increased linear growth velocity during infancy, while those born with normal length but low birth weight did not experience an increased velocity in weight gain.^29^ There are further differences in postnatal growth trajectories between children born at different degrees of prematurity and low birth weight. Together, these distinct growth trajectories have implications for interpreting the literature on associations between intrauterine growth, birth size, postnatal growth, and long-term outcomes, particularly as many studies do not differentiate between these different growth phenotypes. In this article we specify where studies have described a particular growth phenotype, and highlight studies that have not done so.

## CORONARY HEART DISEASE

### Intrauterine Growth/Birth Weight

From the 1980s onwards, several large-cohort studies identified a linear, inverse relationship between birth weight and adult CHD, which formed the basis of what became termed the “fetal origins hypothesis” or “Barker hypothesis.”^35–38^ Birth weight is the most readily available measure of growth in early life, and as the endpoint of intrauterine life, it was often assumed to be a proxy for intrauterine growth. Birth weight is correlated with birth length, and both are predictors of child stunting and adult height.^39^ However, low birth weight is now considered a poor proxy for fetal growth restriction, and different etiologies of low birth weight (whether preterm birth, SGA, or both) may lie on different trajectories of linear growth and cardiovascular disease.^39^ This is a general limitation of the early literature on the fetal origins hypothesis, as most studies did not differentiate by category (preterm birth or SGA) or degree of low birth weight. Two Swedish cohort studies with data on both preterm and SGA found that, from early adulthood onwards, those born SGA had more than 1.5 times the risk of CHD compared with AGA children, after adjusting for gestational age and SEP, whereas gestational age at birth was not associated with CHD (adjusted hazard ratios [95% CI]: 1.64 [1.23, 2.18] and 1.54 [1.15, 2.07], respectively).^40^^,^^41^ However, it has been argued that adjustment for gestational age might introduce collider bias.^42^ A recent register-based cohort study in Sweden and Denmark found that both SGA and preterm birth were associated with CHD in young adults, with less familial confounding for preterm birth than for SGA.^43^

### Postnatal Linear Growth

An association between CHD and short stature in adulthood began to be consistently identified in cohort studies in HICs in the 1980s.^44–52^ A 2010 systematic review of 22 studies, including 3 million individuals from ages 18 to more than 75 years, demonstrated a 1.49 (95% CI: 1.33–1.67) higher risk of CHD among adults in the shortest vs the tallest height categories.^53^ However, adult height is itself a product of a range of factors, including genetics (a potential confounder) and disease-related shrinkage (which is itself a predictor of cardiovascular mortality, and therefore a potential “reverse cause” of the association between height and cardiovascular disease).^54^^,^^55^ Further studies examining the components of attained height identified shorter leg length in adulthood as driving the association of height with CHD.^56^^,^^57^ Leg length is a sensitive marker of nutrition and psychosocial and economic circumstances in early life, because prepubertal linear growth—especially before age 5 years—mainly involves increases in lower limb length. It is therefore a proxy marker of the early-life environment.^58^

Recently, more comprehensive birth cohort data have allowed direct examination of relationships between CHD and length or height at different life stages and/or patterns of linear growth. A systematic review in 2006 found that reduced birth weight and shorter height/lower weight and BMI at 12 months of age were each associated with an increased risk of CHD at ages 53–80 years.^59^ A 2021 systematic review and meta-analysis identified 5 studies investigating associations between height of children and adolescents (from 2 to >18 years of age) and CHD, finding an inverse association of height (as a continuous variable) and CHD incidence from young adulthood to old age; the pooled hazard ratio in the 2021 systematic review was 0.87 per SD increase in height (95% CI: 0.81–0.93), but the degree of heterogeneity was high in the estimates.^60^ In the 2006 review, inadequate consideration of confounding was a frequent source of bias in included studies. In the 2021 review, most studies found associations between height and CHD that persisted after adjustment for SEP.^59^^,^^60^

Twin-pair studies have strengthened the evidence that the association between adult height and CHD is due to environmental factors. A study of monozygotic twin pairs discordant for height and CHD, using data from pooled Danish, Finnish, and Swedish cohorts, found an inverse association between height and CHD death within twin pairs, suggesting the association is driven by individual environmental factors (ie, those not shared with the other twin).^61^ Recently, Mendelian randomization (MR) studies have provided even more robust evidence of a causal relationship between height and CHD. In a European MR study, genetically taller individuals had a 10% lower risk of CHD for every 6.5-cm increase in height—remarkably similar to the risk reduction found in observational studies.^62^ This has been replicated in multiple MR studies, some of which have explored causal pathways through mediation analysis, detailed in the “Mechanisms” section below.^62–64^

There are sex differences in the growth of children who later develop CHD, which could be related to evidence of girls’ lower susceptibility to linear growth faltering in an adverse environment—although this has mostly been studied in low-income settings.^65^ In the 1934–1944 Helsinki Birth Cohort Studies (HBCS), CHD in 45–54-year-old men was related to short stature at ages 1 and 2 years but not to birth length.^36^ After 2 years of age, boys’ height-for-age *z* scores (HAZ) reached a plateau as their BMIs increased. Women with CHD between 45 and 64 years of age were born short but experienced rapid linear growth during infancy, while their BMI *z* scores fell until age 4 and rapidly increased thereafter.^66^^,^^67^ The greatest effect size was seen in girls with short birth length who experienced accelerated linear growth in childhood, which underscores the “growth acceleration” and “capacity load” models, described later.

### Weight-Growth Velocity

Accelerated weight gain—also termed rapid weight gain (RWG) and usually defined as upward centile crossing on a weight growth chart—is more common among those born with a low birth weight or who experienced a period of growth restriction postnatally.^68^^,^^69^ Stunted children, especially those whose socioeconomic environment changes during childhood—such as when migrating from an LMIC to an HIC, or when living in a society undergoing a rapid economic transition—are therefore particularly predisposed to RWG. Weight gain results from both increases in length and soft tissue (fat mass and fat-free mass). In 5 prospective birth cohort studies from Brazil, Guatemala, India, the Philippines, and South Africa (the Consortium of Health-Orientated Research in Transitioning Societies [COHORTS] studies), both RWG relative to linear growth and rapid linear growth relative to weight gain were associated with an increased risk of overweight and hypertension in adulthood.^70^

Observational evidence from 20th-century UK and Finnish (HBCS) cohorts showed that RWG in infancy, at least when following poor intrauterine growth, appears to have a protective effect: low birth weight and slow weight-growth velocity in infancy were associated with CHD.^35^^,^^36^ However, for boys in the HBCS who had a ponderal index (weight [g]/length [cm3] multiplied by 100) of less than 26 at birth, the direction of association reversed after 12 months of age, whereas for those with a ponderal index at birth of more than 26, the pattern of increased risk of CHD relating to low weight gain continued into childhood. Both pathways that ended in the development of CHD were associated with short stature throughout childhood, but these European studies did not differentiate between weight gain independent of vs dependent on length gain. Experimental evidence seems to contradict these findings; trials of different formula milks in SGA infants report higher levels of cardiovascular disease risk factors in childhood following faster infant growth.^71–73^

Together, studies investigating relationships between growth and CHD have revealed 3 important features: First, there is more than 1 pattern of growth that can precede the development of CHD, and this might be modulated by fetal growth; second, CHD seems to be more strongly related to the velocity of growth than anthropometric measures at any particular time point; third, the pace of growth before and after infancy could have differential significance for the development of cardiovascular disease.

### Mechanisms

Recent critiques of the literature on stunting in LMICs have pointed to a lack of evidence for causal relationships between stunting and cardiometabolic disease in LMIC settings, suggesting that studies that found an association between these 2 variables often overlooked a shared set of factors that residually confound their relationship.^74^ The evidence in high-income settings is, similarly, mostly observational and likely to be biased by uncontrolled confounding. However, there are several candidate mechanisms potentially underlying a causal relationship between patterns of early-life growth and adult CHD, or precursors to/risk factors for CHD.

Fetal growth restriction relative to genetically or epigenetically determined growth potential can be followed by growth acceleration in different developmental periods. For example, in a Finnish cohort born in the mid-20th century, rapid catch-up (and sometimes overcompensation) in BMI occurred in mid-childhood, while more recent cohorts show catch-up in infancy.^68^^,^^75^ Such rapid growth is also associated with later cardiovascular risk in observational studies, and this has also been corroborated experimentally in nutritional supplementation trials.^71^^,^^76^ These findings inspired Singhal and Lucas’s^72^ “growth acceleration hypothesis,” which postulated that the combination of fetal growth restriction and rapid growth during infancy or childhood triggers deleterious changes in organ function and substrate metabolism, which can result in cardiovascular disease in adult life. Trial data have demonstrated associations between growth acceleration and endothelial dysfunction, an early stage of atherosclerosis.^77^ This pattern of growth could provide a biological explanation for the social gradient in cardiovascular disease in adulthood, given that socioeconomic differences in childhood growth trajectories have been found to arise largely through inequalities in birth length.^78^

**Figure 1.**
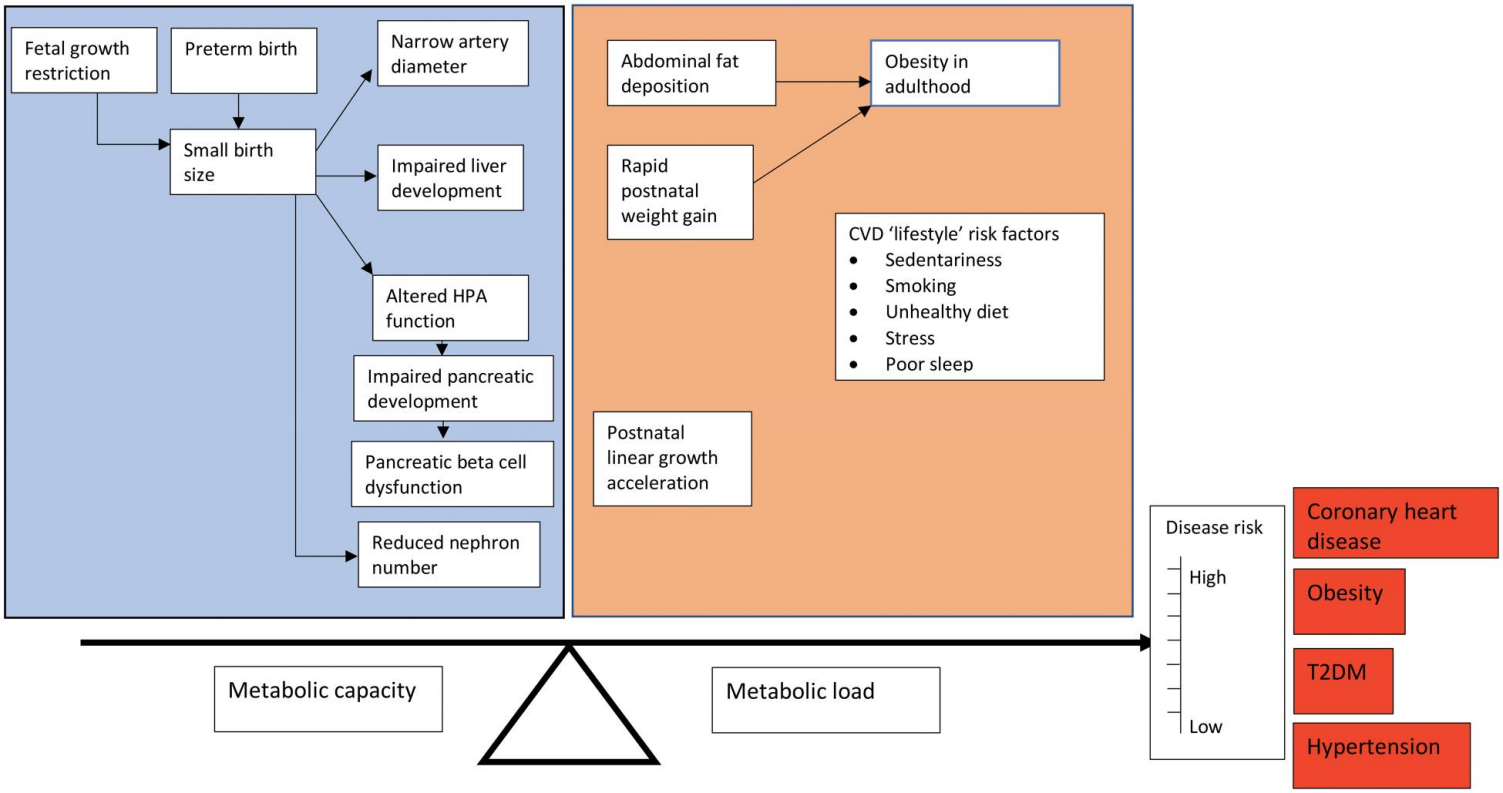
The Capacity-Load Model of Cardiometabolic Disease Risk. Both decreased metabolic capacity (top left-hand box) and increased metabolic load (top right-hand box) independently increase the risk of cardiometabolic disease. Decreased metabolic capacity and increased metabolic load interact, such that the highest risk of disease is among those with decreased capacity and increased load. Note that obesity is both a risk factor for other cardiometabolic diseases, and an outcome in itself. Abbreviations: CVD, cardiovascular disease; HPA, hypothalamic-pituitary-adrenal; T2D, type 2 diabetes

Potential mechanisms underlying these relationships can be expressed using the “capacity load” model of Wells, a conceptual framework describing the interactions between metabolic homeostatic capacity and metabolic load in shaping CHD risk (**Figure 1**).^2^^,^^79^^,^^80^ The concept of metabolic load is analogous to that of “allostatic load,” but metabolic load specifically refers to factors that challenge the body’s capacity for homeostasis of fuel metabolism and cardiovascular function.^2^^,^^81^ Metabolic capacity refers to the body’s ability to maintain homeostasis, and the underlying traits are strongly contingent on growth in early life. High metabolic load increases noncommunicable disease risk, but more so if metabolic capacity is also low. Relevant components of metabolic load include unhealthy diet, sedentary behavior, poor sleep, high BMI and adiposity, smoking, and psychosocial stress. This framework helps to unpack pathways between SEP, growth, and disease across the life course. For example, evidence from HICs has demonstrated a positive relationship between parental/household SEP and intrauterine growth,^13^^,^^82^^,^^83^ while, separately, the association between lower SEP (operationalized using educational level) and CHD has been shown in path analysis to be almost fully accounted for by “traditional” cardiovascular risk factors, including smoking, physical inactivity, and poor diet.^84^ Viewed through the lens of the capacity-load model, poverty therefore predicts both low birth weight/shorter birth length (leading to a reduced metabolic capacity) and “lifestyle” cardiovascular risk factors (collectively constituting a higher metabolic load) that ultimately lead to CHD.

The capacity-load model also helps clarify the apparent contradiction in the evidence described earlier, whereby observational studies found that RWG in infancy is inversely associated with later CHD, whereas the association in the experimental evidence is positive.^35^^,^^36^^,^^71–73^ An interpretation of these studies through the lens of the capacity-load model is that they were describing different, but related, phenomena: The historical cohort studies identified that both low birth weight and poor infant growth were markers of constrained metabolic capacity (eg, poor lean mass accretion, reduced organ function), whereas the trials demonstrated the adverse effects of early elevation in metabolic load (eg, rapid fat accretion).^85^

From an evolutionary perspective, stunting can be viewed through the lens of life history theory.^86^^,^^87^ This theoretical framework assumes that all organisms are under selective pressure to allocate their metabolic resources across 4 competing traits, broadly grouped as “maintenance,” “growth,” “reproduction,” and “defense” against pathogens and social threats. Under the assumption of finite resource availability, this leads to trade-offs whereby, for example, allocating more resources to defense results in fewer resources being available for investment in growth. Inflammation and immune function are costly energy-consuming processes.^88^ Consistent with this framework, there is substantial evidence that greater exposure to infectious disease in early life adversely impacts linear growth.^89^ However, the framework also helps to understand the long-term implications of stunting for cardiometabolic health. Many of the stresses that reduce the availability of metabolic resources for growth also undermine “maintenance,” a term that embraces metabolic homeostatic capacity. In this way, stunting may act both as a broader indirect marker of stresses that impair maintenance and also a direct marker of stresses that impair maintenance by constraining linear growth.

For example, there is evidence for an association between impaired early-life growth and reduced size of adult vital organs. In a study of South Asian women living in the United Kingdom, adult height and leg length were positively associated with volumes of heart, liver, kidney, and spleen measured by magnetic resonance imaging (MRI), as well as muscle mass but not fat mass.^90^ In terms of height components, these associations were stronger for tibial length than for the component of height variability that was independent of tibial length, indicating that poor childhood growth may restrict organ growth, potentially through common hormonal pathways.^90^ Consistent with this, stunting at 2 years was associated with smaller kidney dimensions at 8 years in a cohort of children from rural lowland Nepal.^91^ Moreover, there is observational and experimental evidence that, relative to children of normal height, stunted children have an increased risk of dyslipidemia, potentially resulting from the impact of growth faltering on the developing liver.^62^^,^^92–94^

Mendelian randomization studies have investigated causal pathways between height and CHD. An MR study of people with European ancestry used instrumental variable models that included cardiometabolic risk factors as covariables and found that greater adiposity and lipids, and reduced lung function, contributed to the association between adult height and CHD.^62^ An MR UK Biobank study using mediation analysis found the main mediator of the causal effect of height on CHD to be lung function, while the mediating effect of “traditional” risk factors (including obesity, lipid levels, and blood pressure [BP]) was marginal, and SEP was not a mediator.^64^ These studies corroborate observational evidence in which lung function has almost completely confounded the association between height and heart disease.^62^^,^^95^ The underlying mechanism for this association is unclear, but it is likely due to lung function being a very good marker of another factor. Possibilities include a link with pulmonary hypertension^96^; a shared pathophysiology of reduced elasticity of the cardiovascular and respiratory systems^97^; lung function acting as a marker of narrower structures in general, including both airways and arteries^2^^,^^98^; or childhood factors related to both height and lung development, such as repeated respiratory tract infections—which are associated with lower SEP in childhood.^95^^,^^99^

There is some evidence that stunting might be linked to cardiovascular disease through inflammatory or autoimmune processes. Cardiometabolic disease is a chronic systemic inflammatory pathology, initiated by adaptive autoimmune mechanisms that accompany atherosclerosis.^100^ In settings of high stunting prevalence, short stature is itself associated with systemic inflammation.^101^ One explanation is that this is secondary to chronic intestinal inflammation, termed environmental enteric dysfunction, which is a result of repeated exposure to enteric pathogens.^101^ In high-income settings, where such infections are less common, there is nevertheless evidence of systemic inflammation in children diagnosed with idiopathic short stature, indicating that these children have chronic immune activation, potentially at the expense of homeostasis and growth.^102^ Studies have identified associations between idiopathic short stature and short stature homeobox-containing- (SHOX) gene mutations,^103^ upregulation of the chemokine C-X-C motif chemokine ligand 9 (CXCL9) and the gene/glycoprotein gamma FC receptor Ia / Cluster of Differentiation 64 (FCGR1A/CD64), which is also seen in sepsis and inflammatory bowel disease,^104^ upregulation of interferon-gamma (IFN-γ) gene expression, and higher circulating levels of IFN-γ.^105^ This inflammation may continue into adulthood. In a Japanese study of middle-aged men, the shortest men had a substantially greater odds of having a high circulating leukocyte count than the tallest men (odds ratio [OR]: 1.32; 95% CI: 1.06–1.49).^106^

Analyses from LMICs and HICs have identified an inverse relationship between linear growth and pubertal onset,^107^^,^^108^ which is important because there is an association between earlier pubertal timing and cardiometabolic disease in adulthood.^109–111^ Several studies of data from children who have migrated from LMICs to HICs through adoption have found that catch-up linear growth is also associated with pubertal development, but seemingly only if it is preceded by intrauterine growth restriction (IUGR).^112–115^ The pace of growth acceleration is correlated with the rate of pubertal development; a study of girls adopted from India to Sweden found that the shortest children with the fastest linear growth had the earliest menarche.^116^ These effects are likely to be transgenerational. In a UK cohort, earlier age at menarche was associated with significantly shorter adult height, but in the next generation, children of women who had earlier menarche had significantly faster gains in both height and weight in early life.^117^ While further research is required, the findings from these studies suggest possible biological mechanisms for social and ethnic inequalities in health outcomes, given findings in multiple settings of substantial social and ethnic differences in linear growth patterns and puberty.^118–120^

## OBESITY/BODY COMPOSITION

Investigating relationships between obesity and other measures of growth or body composition throughout the life course is complex for several reasons. First, greater weight during childhood seems to drive faster linear growth, making it difficult to disentangle the effects of obesity and linear growth.^121^ Second, the timing of the outcome measurement is important: obesity in childhood is correlated with obesity in adulthood, but not all obese children become obese adults, and it is not clear that childhood obesity is an independent risk factor for cardiovascular disease.^122^ Third, there are inconsistent measures of childhood obesity in the literature, making comparison between cohorts challenging.^123^ Fourth, BMI is a poor proxy for body fat, and few studies deploy methods to distinguish lean and fat mass.^124^ Fifth, if stunting is defined by HAZ and obesity by BMI, then there is a risk of autocorrelation, due to the fact that height appears in both measures.^125^ Last, patterns of growth (linear, weight, and BMI) and body composition are distinct in different settings globally, and may themselves reflect transgenerational effects of different histories of poverty and phenotypes of malnutrition, including stunting, wasting, and obesity. Given the magnitude of the literature in this area, in the following section we consider separately (1) studies from LMICs and HICs and (2) data relating to child/adolescent and adult outcomes.

### Linear Growth in LMICs

Several cross-sectional studies—and some longitudinal studies—from the 1960s onwards from LMICs including Peru,^126^ Brazil.^127^ and Jamaica^128^ identified a positive association between stunting and obesity in childhood.^127^^,^^129–132^ However, these cross-sectional studies may be subject to bias from imprecise height measurements, whereby an underestimation of measured height can result in a misclassification of BMI and obesity.^125^ Furthermore, most subsequent longitudinal studies in LMICs contradicted these earlier findings. In Jamaica, children who were stunted between 9 and 24 months had lower BMI and percentage body fat by age 11 years,^133^ and studies in other LMICs including Bolivia,^134^ Brazil,^135^^,^^136^ Senegal,^137^ Nepal,^91^ and Guatemala^138^ have had similar results. However, in Jamaica, the subgroup of stunted children who experienced greater accelerated linear growth in adolescence had a higher BMI at age 17 years.^139^ Other cohorts followed longitudinally have found no association between stunting and BMI in childhood,^140^ although many of these longitudinal studies have found that stunted children with lower BMIs have a greater propensity to a central distribution of fat.^133^^,^^137^^,^^141^

More recent longitudinal studies in LMICs, including the COHORTS studies in Brazil, Guatemala, India, the Philippines, and South Africa, have identified positive associations between child height and BMI from childhood through adulthood,^139^^,^^142–144^ although in these studies, linear growth was usually more strongly associated with lean mass than with fat mass or abdominal circumference.^70^^,^^143^

### Linear Growth in HICs

#### Child and Adolescent Obesity

Several studies in HICs in the 1980s identified cross-sectional relationships between stunting, accelerated linear growth, and obesity (or body composition) in childhood or adolescence.^145–147^ However, there are conflicting findings from longitudinal studies, even in economically comparable settings at similar time points. Among children born in the Netherlands between 2003 and 2005, birth weight, accelerated linear growth, and weight gain were all positively associated with both fat mass and fat-free mass,^148^ but among children born in France in 1992, there was no evidence that birth length and height growth velocities were associated with BMI or body composition.^149^ These differences might be accounted for by evidence for relationships between both high and low birth weight with obesity in childhood and adulthood.^150^^,^^151^ This implies that there are at least 2 pathways linking fetal growth with later body composition.^152^ It has been suggested that this U-shaped association sometimes continues into adulthood, and is related to 2 distinct mechanisms (discussed in the “Mechanisms” section below).^153^

#### Adult Outcomes

A 2012 systematic review and meta-analysis of approximately 650 000 individuals found that low birth weight (<2.5 kg) was associated with a decreased odds of overweight in adulthood (OR: 0.73; 95% CI: 0.64–0.85) and that high birth weight (>4 kg) was associated with an increased odds of overweight (OR: 1.60; 95% CI: 1.45–1.77), when compared with children of normal birth weight. The review found that this observational evidence was overall of high quality, with a low risk of bias and confounding.^154^ In a study of participants in the UK Biobank, both observational and MR approaches identified a positive association between birth weight and BMI in adulthood.^155^ Most observational studies in HICs have found a positive association between height in childhood and adult BMI or body composition.^156–160^ However, associations between early-life growth and later obesity are complicated by changing relationships between height and body composition throughout the life course. In a German study, while height and BMI were positively associated with each other in children and adolescents, the opposite was true in adulthood.^18^ Similarly, a Swedish study demonstrated an association between BMI gain and height gain in childhood, both of which were associated with an earlier onset of puberty, which, in turn, reduced height gain in adolescence.^121^ Taller, obese children can experience premature skeletal maturation, limiting their final height, so that they become adults with short or average height.^161^ Consequently, height measured at 1 time point can be misleading—and this is further complicated because the strongest positive associations between height and obesity or cardiovascular disease are often seen among tall children with the shortest birth lengths.^66^^,^^68^ Therefore, although height and obesity were positively correlated at the time of height measurement in many of the studies cited above, in some cases the association would have been in the opposite direction if measured during childhood, and in the same direction if measured at birth.

Other cohort studies in HICs have found persisting relationships between short height and obesity throughout life.^162^^,^^163^ A 2019 review identified 11 studies investigating linear growth in childhood and later body composition, finding that taller child height (but not faster linear growth velocity) was associated with increased lean mass in adulthood, whereas other measures of obesity or body composition in adulthood did not have consistent associations with linear growth.^164^

### Weight-Growth Velocity

#### High-Income Countries

##### Child/adolescent outcomes

Several studies in HICs have investigated relationships between RWG and measures of body composition later in childhood and adolescence. While most studies found a positive correlation between RWG and later obesity, there were conflicting findings in relation to the timing of the RWG. Some studies (in Sweden, the United Kingdom, and the United States, for example) have identified the entire duration of infancy or even up to 2 years of age as periods of RWG affecting later body composition,^165–167^ but others (in France, the United Kingdom, the Netherlands, and the United States) have found that the associations disappeared for RWG after 4,^168^ 6,^149^^,^^169^ 8,^170^ or 9 months.^171^ Other studies have found that, in addition to the first 6 months after birth, RWG after 2 years^149^ or 3^172^ years also had a relationship with childhood or adolescent obesity. These findings support the concept of context-specific “critical windows” for intervention in the promotion of linear growth.^173^

However, these studies exemplify the methodological differences that complicate comparisons of studies, as each used a different cutoff in parameterizing RWG. The first UK study cited above defined RWG as weight gain over the 90th centile, without considering length gain, and did not control for potential confounders^165^; the first US study cited defined RWG as crossing more than 2 weight-for-length centiles, and controlled for age, sex, period, and race/ethnicity^166^; the Swedish study used a change in weight *z*-score correlation coefficient, and adjusted for sex, age group, sexual maturation, and SEP^167^; the second US study cited used weight gain in grams per month but did not measure length gain, and adjusted for birth weight, gestational age weight at 12 months, sex, race/ethnicity, first-born status, and maternal BMI and education^168^; and finally, the second UK study cited used change in weight *z* score, did not measure length, and controlled for pubertal timing, parental anthropometry, SEP, and ethnicity.^171^ Recent studies have used more specific measures for RWG. A US study found that obesity at 6–11 years of age was more strongly related to rapid accrual of fat mass during infancy rather than total mass, but the study was not adequately powered,^170^ and the study in the Netherlands cited above used an increase in fat mass percentage *z* score of more than 0.67, adjusting for sex, gestational age, age, and length gain, but did not measure outcomes beyond 2 years of age.^169^

##### Adult outcomes

Systematic reviews have identified associations of RWG in infancy and early childhood and subsequent obesity in adulthood.^174^^,^^175^ Studies of RWG among children born preterm or SGA are not always included in these systematic reviews but are nonetheless highly relevant to the stunting literature because, even in HICs, these children are more likely than others to have short birth lengths, to be stunted until at least 24 months, and to continue to be short through adulthood.^173^ In a Spanish study, SGA children gained relatively more abdominal fat and less lean mass than children born AGA.^176^ A study of preterm infants in the Netherlands found that RWG in the first 6 months of postnatal life was positively associated with body fat percentage and waist circumference in early adulthood.^177^ However, other studies have not found such relationships and a systematic review reported overall limited evidence among preterm children.^33^

#### Low-Income Countries

There is also evidence of relationships between RWG and later obesity in LMICs. In studies from several LMICs, growth patterns between 0 and 1 year of age and over 2 years predicted body composition in adolescence and adulthood, but RWG between 0 and 1 year more strongly predicted later lean mass, while after 2 years it was associated with fat mass, and the overall evidence was stronger for the period after 2 years than for infancy.^70^^,^^143^^,^^178–182^

### Mechanisms

While there is conflicting evidence from different contexts in relation to stunting in childhood and body composition in adulthood, there are some mechanistic explanations for positive associations between stunting and obesity. Differences in respiratory quotient (RQ), the ratio of the volume of carbon dioxide expired to the volume of oxygen consumed during respiration, has been identified as a mechanism for increased weight gain. A higher RQ indicates a low ratio of fat to carbohydrate oxidation.^183^ In South Korea, children with an HAZ of 1.0 or less and an HAZ of 2.0 or less had a significantly higher RQ than children of normal height (β = 0.036 and 0.060, respectively; *P* = .018 or .016), independent of age, gender, and body composition.^184^ These results suggest that early stunting could predispose a child to metabolic adaptations that favor fat deposition, especially if, following a period of growth faltering, they subsequently receive increased nutrient intake. Considering this from an evolutionary perspective of trade-offs between homeostasis, growth, reproduction, and immune function, stunted children will experience the biggest returns on reproductive fitness by enhancing immune function and reproductive capacity, at the expense of homeostasis (due to shorter life expectancy, they may never pay the full costs of elevated CHD risk). Adipose tissue—especially when deposited centrally—is immunologically active; hence, abdominal fat stores can be considered to have a defensive role in stunted individuals, who are at higher risk of death from infectious disease.^5^^,^^185^ It is possible, therefore, that when stunted children are exposed to an obesogenic environment, they are more likely to deposit fat centrally, as this physiological tactic may have different Darwinian fitness benefits and costs compared with non-stunted children.

The hypothesis that the shift away from fat metabolism and towards carbohydrate metabolism could result in increased deposition of fat was tested in a study in Siberia, which found that women with an HAZ of 1 or less had significantly lower fasting fat oxidation levels than taller women. Reduced fat oxidation in this cohort was correlated with percentage body fat, serum triglyceride levels, and serum leptin levels.^186^ However, a study of children in Cameroon failed to replicate this, finding conversely that a high birth weight predicted poor fat oxidation.^187^

## TYPE 2 DIABETES

### Intrauterine Growth/Birth Weight

Systematic reviews in 2003, 2008, 2019, and 2019^188–191^ have corroborated the findings, in a UK cohort, that the risk of developing T2D was over 10 times greater among individuals whose birth weight was 2.95 kg or less compared with those born with a birth weight of more than 4.31 kg.^192^ The development of T2D in adulthood has also been associated with exposure to famine during pregnancy, supporting a fetal origins hypothesis.^193^ The 2003 and 2018 systematic reviews did not distinguish between etiologies of low birth weight.^189^^,^^191^ The 2008 systematic review identified 10 studies in which it was possible to differentiate by preterm status, and restriction to preterm birth did not substantially alter the association.^188^ The 2019 systematic review included a meta-analysis that found a dose–response relationship between birth weight and T2D risk, but cited only 1 study that differentiated term AGA and preterm SGA infants.^190^ Evidence for an association between T2D and the intrauterine environment was supported by twin studies in the 1990s, which found that, in monozygotic and dizygotic twin pairs discordant for impaired glucose tolerance, the twins with impaired glucose tolerance (in both early and late adulthood) had lower birth weights.^194^^,^^195^ A recent MR study strengthened the evidence for a causal association, finding over twice the odds of T2D for a 1-SD decrease in birth weight (OR: 2.10; 95% CI: 1.69–2.61). Concerningly—given the increasing prevalence of childhood obesity in HICs—recent evidence suggests that children with low birth weight who are obese in childhood are at higher risk of insulin resistance than children who do not become obese.^196^^,^^197^

### Postnatal Linear Growth

In the 1924–1933 HBCS, the incidence of diabetes in adulthood was inversely associated with birth length, but stunted infants who went on to develop diabetes experienced accelerated linear growth between birth and 15 years of age.^198^ The OR for the development of diabetes was 1.44 (95% CI: 1.22–1.72) for each *z*-score increase in height between 7 and 15 years of age. The effect of accelerated linear growth was modified by birth weight: The corresponding ORs were 1.83 (95% CI: 1.37–2.45) for those with birth weight less than 3 kg and 1.25 (95% CI: 1.06–1.48) for those with birth weights greater than 3 kg (test for interaction *P* = .02).^198^ Analysis of the later 1933–1944 HBCS revealed 2 pathways of growth that predicted later diabetes.^199^ Among those with birth weights greater than 3.5 kg, slow linear growth from 0 to 3 months predicted diabetes, and this pattern of growth potentiated the effect of later weight gain, whereas among those with birth weights less than 3.5 kg, the rate of infant growth was unrelated to later diabetes incidence.^200^ Cohort studies in other European countries have similarly found that birth length and linear growth in childhood or adolescence have relationships with T2D in opposite directions.^198^ Conversely, numerous studies have identified an inverse relationship between adult leg length and diabetes risk, suggesting that reduced prepubertal growth is associated with later diabetes. Together, these studies suggest that diabetes is associated with periods of reduced growth in early life (constraining capacity), followed by accelerated growth (elevating load) later in childhood and adolescence. However, MR studies have identified causal relationships between diabetes and adult height, but not infant length.^201–206^

The data from LMICs are mixed. Observational data from Mexico identified associations between stunting and significantly higher levels of insulin and glucose, especially for those with higher BMIs in adulthood.^207^ However, in the COHORTS studies, linear growth was unrelated to either adult plasma glucose concentration or dysglycemia.^70^ The data on relationships between attained adult height and diabetes (both T2D and gestational diabetes mellitus) are also mixed: Most studies in LMICs have found an increased risk of diabetes in shorter adults,^208–210^ but a multi-country analysis of individual-level data identified no relationship.^211^

### Weight-Growth Velocity

Observational data have demonstrated an association between RWG and pathophysiological precursors to diabetes. A study investigating insulin sensitivity and secretion among participants of the UK Avon Longitudinal Study of Parents and Children (ALSPAC) cohort study found that lower insulin sensitivity at age 8 years was associated with RWG from birth to 3 years (defined as a gain in weight *z* score >0.67), but mostly unrelated to birth weight. In contrast, the association between lower compensatory insulin secretion and birth weight was independent of postnatal weight gain, indicating 2 separate pathways to diabetes pathogenesis in this cohort: the first relating to RWG and mostly unrelated to intrauterine growth and the second related to factors in utero.^212^

Nutritional supplementation trials corroborate these findings of different postnatal growth and metabolic trajectories that are dependent on intrauterine growth. In Guatemala, nutritional supplementation of stunted children until age 2 years was associated with reduced odds of T2D (OR: 0.46; 95% CI: 0.21–0.97), but with increased odds of obesity at ages 37–54 years.^213^ Conversely, in the United Kingdom, insulin resistance—measured by fasting 32–33 proinsulin concentration—was greater in adolescents who had been born preterm and fed a nutrient-enriched diet than in preterm infants given a lower-nutrient diet (mean difference: 20.6%; 95% CI: 5.0%–36.3%). Insulin resistance was independently associated with greater weight gain in the neonatal period.^76^ Unfortunately, birth weight and length were not available in the Guatemala trial, and postnatal linear growth patterns were not available in the UK trial, but the differences between these trials could be explained by data revealing the contrasting responses of SGA and AGA infants to increased nutrition postnatally. In a Danish study involving SGA and AGA infants, RWG—defined as an increase in weight SD score between 0 and 3 months—was independently associated with insulin resistance at 17 years of age only among those born SGA, whereas for children born AGA this association was mediated by later adiposity.^214^

### Mechanisms

Several studies have identified an inverse relationship between birth weight and insulin resistance but a positive relationship between postnatal accelerated linear growth and insulin resistance.^215–217^ These relationships in opposite directions could be explained by the “thrifty phenotype” hypothesis, whereby brain growth is selectively protected at the expense of other organs, including the pancreas.^218^ Intrauterine growth restriction appears to have an impact on pancreatic development, a theory that is supported by studies that have found children who experienced IUGR to have higher plasma 32–33 split proinsulin concentrations, a measure of beta-cell dysfunction.^216^ Ongoing impaired growth during the infant period might also impair pancreatic development, as the pancreas continues to develop during infancy.^219^

Insulin-like growth factor 1 (IGF-1), a polypeptide produced by the liver, is related to both childhood growth and diabetes in adulthood, and as such, could be a mediator between the two.^212^^,^^220–222^ Several studies have found decreased plasma levels of IGF-1 among stunted children, which could be due to either suppression of IGF-1 by chronic, low-grade inflammation,^223^^,^^224^ or to the effects of malnutrition during hepatic development, providing a biological mechanism for the “thrifty phenotype.”^192^

Programming of the hypothalamic-pituitary-adrenal (HPA) axis in early life by fetal or infant growth may also alter the risk of developing T2D.^225^ Cortisol levels are increased in growth-restricted fetuses, which reduces birth weight and influences the HPA axis.^226^ Through epigenetic changes to the glucocorticoid receptor gene promoter, altered HPA function can impair development of glucocorticoid-responsive tissue, such as the pancreas.^227^

It has also been suggested that there could be shared genetic factors that underlie low birth weight and T2D. First proposed in 1998, this was termed the “fetal insulin hypothesis.”^228^ Initially this was largely theoretical, but recent advances in genome-wide association and MR studies have identified shared genetic determinants of low birth weight and diabetes.^229^^,^^230^

## HYPERTENSION

### Intrauterine Growth/Birth Weight

Several reviews published between 1996 and 2006 found that birth weight is inversely related to BP in children and adults,^231–234^ findings that are supported by more recent data from large European birth cohort studies.^235^ There are mixed findings with regard to the etiology of low birth weight, with most studies included in the 2000 systematic review finding no association between gestational age and systolic BP (SBP), suggesting that the association is with SGA rather than preterm status.^232^ A 2012 systematic review found higher SBP (pooled estimate: 2.5 mmHg; 95% CI: 1.7–3.3) among former preterm or very-low-birth-weight infants (<1500 g) than among term infants, after adjustment for SEP and adult-attained height or weight, but did not differentiate between etiology of low birth weight.^236^ A 2023 systematic review found slightly higher BP (<5 mmHg) in young adults born preterm than those born at term.^237^ Overall, however, the quality of evidence is low.^232^^,^^235^^,^^237^^,^^238^ Recently, MR studies did not identify a causal relationship between low birth weight and adult hypertension.^155^^,^^239^ This also suggests that the causal pathway between low birth weight and other cardiovascular diseases, including CHD, is not mediated by hypertension.^155^

Lower birth weight was associated with elevated BP in observational evidence from the middle-income-country COHORTS studies, but only after adjustment for adult height and BMI, implicating a moderating effect of “catch-up” growth in weight and height.^240^^,^^241^ Studies in China and Australia have had similar results.^242^^,^^243^ Generally, adults at the highest risk of elevated BP are those with low birth weight and a high BMI in adulthood.^240^

### Postnatal Linear Growth

Cohort studies in HICs have identified inverse associations between child height and adult blood pressure—of up to a nearly 30-mmHg difference between the shortest and tallest children in a UK cohort study of children born in the 1930s, after adjustment for a wide range of potential anthropometric and socioeconomic confounders.^241^^,^^244^ The middle-income-country COHORTS studies found, conversely, that weight, height, faster linear growth, and BMI in early childhood were all positively associated with SBP.^70^^,^^240^

Differences in findings between studies might, again, be explained by both the timing of measurement and the pattern of growth. Two distinct pathways of childhood growth among hypertensive adults have been identified. On 1 pathway, infants are born with low birth weight and short birth length and then experience a rapid weight and linear growth acceleration postnatally (while adjustment for gestational age did not substantially change the estimates, and there was no independent association of BP with gestational age).^245^^,^^246^ On another pathway, low birth weight and short birth length are followed by persistently slow linear growth so that children remain short into adolescence^247^ and adulthood.^248^ These studies are corroborated by data indicating an association between hypertension and leg length.^249^ The most recent systematic review of studies investigating associations between birth size, postnatal growth, and adult BP identified 3 studies that demonstrated associations between hypertension and this first pathway of growth,^232^ supporting the conceptual model of decreased capacity in antenatal and early postnatal life, and increased load in later life stages. A recent systematic review of 18 studies found an inverse relationship between height and hypertension in adulthood, but there was a high degree of heterogeneity.^250^

### Weight-Growth Velocity

Evidence from a 2000 systematic review suggests that RWG in infancy and childhood is positively associated with hypertension, which was true for RWG defined by both the difference between birth weight and adult weight/BMI and the difference between birth weight and adult height.^232^ One study included in the review found that the strongest associations were between those who had lower birth weights and greater height in adulthood.^232^ Several analyses from LMICs and HICs have been published since this review, mostly with corroboratory findings.^70^^,^^73^^,^^251–253^ However, many of these studies have found stronger associations with growth in mid-childhood than in infancy, having found either no evidence for an association between RWG from 0 to 2 years of age and hypertension,^252^ or associations had small effect sizes,^70^ or, in some cases, RWG was associated with lower BP.^254^^,^^255^

### Mechanisms

The evidence for a causal relationship between hypertension and stunting at any age is weaker than that for other cardiovascular outcomes, and there appears to be no causal relationship with birth weight. However, studies in animals and humans have found that IUGR is associated with the development of fewer nephrons by term, which is particularly important because there is no postnatal cell replication in the kidney.^256^ The capacity-load model could therefore explain consistent findings in the literature of associations between hypertension and growth phenotypes involving a combination of low birth weight and postnatal growth acceleration, as this could be due to a higher rate of perfusion per nephron, resulting in glomerular sclerosis and hypertension.^256^

## COGNITIVE DEVELOPMENT

Overall, there are many more contemporary studies investigating associations between child growth and cognition in LMICs than HICs and the evidence in HICs—which is mostly historical—is conflicting.

### Low-and-Middle-Income Countries

Data from the Young Lives Study in Ethiopia, India, Peru, and Vietnam have demonstrated positive associations of linear growth in infancy and cognition from childhood to adolescence.^257–261^ A recent meta-analysis of the COHORTS studies found associations between linear growth from 0–2 years of age and both educational outcomes and intelligence quotient (IQ) in adulthood.^262^ Similar findings have emerged over the past 15 years from other LMIC cohorts.^263–273^ A 2015 meta-analysis of studies from 29 LMICs found that each unit increase in height SDS for children over 2 years old was associated with a 0.09 SD (95% CI: 0.05–0.12) increase in cognitive score.^268^

### High-Income Countries

Cross-sectional studies from HICs have identified positive relationships between height and educational outcomes at school age.^274–276^ Longitudinal associations between growth and cognition have also been described in HICs, although most of these data are from mid-20th century UK cohorts.^277–279^ The 1946 British Birth Cohort Study identified positive relationships between height and cognitive scores between 8 and 24 years of age, with the relationship strengthening at older ages.^277^ In a 2004 review of studies from HICs, although most children with short stature had cognitive scores in the normal range, stunted children overall had significantly lower cognitive scores, academic achievement, and visual-motor ability than others.^280^ However, the overall quality of the evidence in studies identified was low.^280^ Some more recent birth cohorts in HICs have found no association between childhood height and cognitive measures,^281^^,^^282^ and this could be related to findings, from Denmark, of a weakening association between height and IQ in successive cohorts.^283^ However, a recent analysis of children participating in the Millennium Cohort Study (MCS; a study of children born in the 21st century in the United Kingdom) found that children who were stunted at age 3 years had language scores between ages 3 and 11 years approximately 0.25 SDs below those with normal height at 3 years.^284^

### Timing of Growth Faltering

There are clear associations between the timing of linear growth and various neurodevelopmental domains, although most relevant studies are from LMICs. In these settings, while intrauterine and infant growth are significantly associated with downstream cognitive scores and academic outcomes,^285^^,^^286^ linear growth beyond the age of 2 or 3 years appears to be associated with a significantly reduced effect size,^268^ or not associated with these outcomes.^262^^,^^267^^,^^287^^,^^288^

### Mechanisms

There are several potential pathways between stunting and child development, although mechanistic research in this area is limited. Furthermore, much of the research relates to stunting as a manifestation of undernutrition, including micronutrient deficiencies, or to relationships between stunting, cognition, and recurrent or chronic enteric infections. In HICs it is likely that these factors explain less of the growth faltering experienced by children living in poverty.

However, some proposed mechanisms are more likely to be common to stunted children globally. The strong association between preterm birth and learning disability could explain some of the correlation between stunting and cognitive or education outcomes, as a proportion of stunting is attributable to preterm birth.^289^ Both preterm birth and SGA, despite relative brain sparing, are associated with smaller brain volume throughout life and with lower cognitive scores, but the evidence in HICs for a causal association between brain volume and impaired cognition and education outcomes is weak.^290–292^ Insulin-like growth factors, peptides whose function includes the regulation of growth, target areas of the brain (such as the hippocampus) that are responsible for learning and memory.^293^ Insulin-like growth factors interact with thyroid hormones and cortisol, both of which are linked to linear growth, weight, and cognition, although how these might mediate relationships between stunting and neurodevelopment is unclear.^294–296^

In addition to impaired cognition, children with stunted growth often have deficits across social, emotional, and motor development.^297^^,^^298^ Stunting is also often related to various behaviors, including apathy and reduced exploratory behavior, and stunted children have been found to experience substantially more anxiety and depression and lower self-esteem.^299^ It is possible that these psychological factors, in addition to global developmental impairment, interact to inhibit cognitive and psychosocial development—for instance, through limiting a child’s independent play and social interaction. As a marker of poverty, stunting is also likely to be (non-causally) associated with neurocognitive development through relationships between adverse childhood experiences and structural differences in brain development^300^^,^^301^ and epigenetic changes and calibration of the physiological response to stress.^302^

## CONCLUSION

Stunting, cardiometabolic disease, and neurodevelopment are complex phenomena, rooted in multiple exposures across the life course. Relationships between childhood growth and downstream chronic disease and neurocognitive outcomes are challenging to interpret because the evidence from high-income settings is mostly observational, retrospective, and is often based on historical data. Relationships between early-life growth and chronic disease outcomes are not constant throughout the life course, and therefore conflicting results from different cohorts—even in economically comparable settings at similar time points—are sometimes explained by differences in the age of measurement of dependent and outcome variables. However, in many cases, these differences are difficult to account for, and likely reflect a combination of methodological differences, uncontrolled confounding, distinct environmental exposures, transgenerational effects, and a variable potential for accelerated growth in different settings globally.

The strength and consistency of the independent longitudinal associations between linear growth and several long-term deleterious outcomes in multiple study types and across diverse settings and time points identified in this review are striking. Nutrition supplementation trials, twin studies, and MR studies have strengthened the evidence for a causal relationship between growth and cardiometabolic disease; further studies investigating causal pathways will help guide how and when interventions might prevent long-term sequelae. In addition, growth monitoring is relatively straightforward and is already established, although often underutilized, in most HICs. Further research should be directed at the potential for using height and growth velocity as markers for identifying children at increased risk of lifelong decrements in educational or cardiometabolic health outcomes, who could benefit from early intervention.
